# Comparative study of the functional properties of lupin, green pea, fava bean, hemp, and buckwheat flours as affected by pH

**DOI:** 10.1002/fsn3.143

**Published:** 2014-10-07

**Authors:** Vassilios Raikos, Madalina Neacsu, Wendy Russell, Garry Duthie

**Affiliations:** 1 Natural Products Group, Rowett Institute of Nutrition and Health, University of AberdeenAB21 9SB, Scotland, UK

**Keywords:** Emulsion, flour, foaming properties, pH, protein functionality

## Abstract

The demand for products of high nutritional value from sustainable sources is growing rapidly in the global food market. In this study, the effect of pH on the functional properties of lupin, green pea, fava bean, hemp, and buckwheat flours was investigated and compared with wheat flour. Functional properties included solubility, emulsifying and foaming properties, gelling ability, and water holding capacity (WHC). All flours had minimal solubility at pH 4 and their corresponding values increased with increasing pH. Emulsifying properties were improved at pH 10 for all samples and emulsion stability showed a similar trend. Increasing pH in the range 4–10 enhanced the foaming properties of the flours, particularly buckwheat and hemp. Wheat, green pea, buckwheat, and fava bean were more capable of forming firm gels compared with lupin and hemp, as indicated by least gelling concentrations (LGCs). The ranking of the water binding properties of the different types of flours were lupin>hemp>fava bean>buckwheat>green pea>wheat. Results indicate that underutilized flours from sustainable plant sources could be exploited by the food industry as functional food ingredients or as replacements of wheat flour for various food applications. Depending on the application, flour functionality may be effectively tailored by pH adjustment.

## Introduction

Wheat flour is the main ingredient of most bakery products. Moreover, its versatile physicochemical properties are exploited as a functional ingredient in the manufacture of many food products across the world (Aguilera et al. 2011). However, price fluctuations on the commodities market can be problematical for countries which depend on importation to meet the demand for wheat flour (Noor Aziah and Komathi 2009). Furthermore, predicted demographic and environmental changes suggest an increasing need to develop healthy foods using raw materials from sustainable, underutilized sources. Protein malnutrition is prevalent in developing and underdeveloped countries due to the limited availability of animal protein (Bhat and Karim 2009). Economic, environmental, and health-related issues are therefore the main driving forces aiming to identify alternative plant sources as food ingredients (Sánchez-Vioque et al. 1999).

The applicability of alternative plant flours as wheat flour substitutes or functional ingredients in food products depends to a large extent on their protein composition. Protein-related functional properties including water and fat binding, emulsifying properties, foaming capacity, and gelation impart beneficial qualities which facilitate utilization in food manufacturing systems (Kinsella 1976; Kaur and Singh 2005). Protein functionality depends on not only intrinsic factors such as molecular size and structure but also on extrinsic factors including interactions with other food components, pH, ionic strength, heat treatment, and other processing conditions (Kinsella 1976; Moure et al. 2006). Consequently, understanding and controlling protein functionality of flours derived from different plant sources is a prerequisite for the development of economically viable, high-demand products.

Nutritional guidelines emphasize the importance of high-fiber diets for prevention of various health disorders (Anderson et al. 1994). High protein, fiber rich, and potentially sustainable alternatives to wheat include lupin, green pea, fava bean, hemp, and buckwheat. Furthermore, the acidity of foods systems varies depending on the ingredients and the processing methods. Acidification, for instance, is an important step in the manufacture of products such as mayonnaise and salad dressings and pH may be as low as 4.5 (Smittle 2000). As the usefulness of flours from these plants as ingredients for new food products and formulations depends on protein functionality, the present study has systematically compared the effect of pH on their functional properties in relation to commercial wheat flour. The main objective of this study is therefore to provide essential information useful for the incorporation of flours from alternative plant sources into food systems.

## Materials and Methods

### Materials

Strong white flour was purchased from Tesco (Chesthunt, UK), buckwheat flour from Arrowhead Mills, Inc. (Boulder, CO), hemp flour from Yorkshire Hemp Ltd. (Driffield, UK), fava bean flour from The Barry Farm (Wapakoneta, OH), green pea flour from Bob's Red Mill Natural Foods (Milwaukie, OR), and lupin flour from Terrena Lup Ingredients (Martigne Ferchaud, France). The macronutrient composition of all flours sourced from the corresponding product labels is presented in Table1. Rapeseed organic oil was obtained from the local supermarket (Tesco). Sodium dodecyl sulfate (SDS) and Protein Quantification kit were supplied by Sigma-Aldrich Corp. (Dorset, UK). Disodium hydrogen orthophosphate anhydrous were provided by Fisher Scientific International Inc. (Loughborough, UK) and sodium dihydrogen orthophosphate 1-hydrate was purchased from British Drug Houses Chemicals (Philadelphia, PA). Laemmli sample buffer, Tris/Glycine/SDS running buffer, Coomassie Brilliant Blue R-250 staining and destaining solutions, 2-mercaptoethanol, prestained SDS-PAGE standards (broad range), and precast gels were purchased from Bio-Rad Laboratories Inc. (Hemel Heampstead, UK). All reagents used were of analytical grade.

**Table 1:** Macronutrient composition of the flours.

Flour type	Protein (g kg^−1^)	Total carbohydrate (g kg^−1^)	Fiber (g kg^−1^)	Total fat (g kg^−1^)
Wheat	126	685	31	14
Lupin	400	100	350	100
Green pea	267	600	267	0
Fava bean	300	633	267	17
Hemp	279	507	220	89
Buckwheat	167	667	200	50

### Protein solubility

The protein solubility was determined according to the method of Morr et al. (1985) and corresponded to the dissolved protein fraction relative to the total protein content. Plant flours (50 g kg^−1^) were suspended in 10 mmol/L Na_2_HPO_4_–NaH_2_HPO_4_ buffer (pH 4.0, 7.0, and 10.0) for 30 min at room temperature. The flour dispersions were centrifuged at 11,337*g* for 10 min and the protein content of the supernatant was determined by the Bradford (1976) method at 600 nm. Calibration of the assay was performed with standard bovine serum albumin solution. Protein solubility was calculated using the following equation:

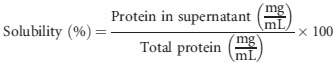
(1)

### Emulsion preparation

Plant flours (50 g kg^−1^) were suspended in 10 mmol/L Na_2_HPO_4_–NaH_2_HPO_4_ buffer (pH 4.0, 7.0, and 10.0) for 30 min at room temperature prior to the addition of rapeseed oil. Oil-in-water emulsions, 200 g kg^−1^, were prepared using an Ultra-Turrax T18 homogenizer (Janke & Kunkel; IKA Instruments, Staufen, Germany) set to a speed of 12,000 rpm for 2 min.

### Emulsifying activity and stability indices

Emulsifying activity (EAI) and stability (ESI) indices of flour samples were determined as described by Pearce and Kinsella (1978). An emulsion sample (50 *μ*L) was taken from the bottom of the tube immediately after homogenization and diluted in 7.5 mL of 10 mmol/L Na_2_HPO_4_–NaH_2_PO_4_ buffer containing 0.1% SDS and then vortexed for 5 sec. An aliquot of this mixture was taken after 10 min of static storage at room temperature. Sample absorbance was measured at 500 nm by means of a Pye Unicam UV-4 UV-VIS scanning spectrophotometer (Spectronic Camspec Ltd., Leeds, UK) using plastic cuvettes (0.01 m path length). EAI and ESI values were calculated using the following equations:

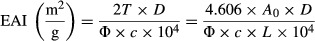
(2)

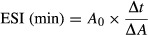
(3)where *T* is turbidity, *D* is dilution factor (× 150), *Φ* is the volume fraction of the dispersed phase (0.2), *c* is the weight of the protein per unit volume before the emulsion is formed (g/mL), *A*_0_ is the absorbance of the diluted emulsion immediately after homogenization, *L* is the path length of the cuvette, Δ*A* is the change in absorbance between 0 and 10 min, and Δ*t* is the time interval (10 min).

### Creaming stability measurements

Following homogenization, 10 mL of emulsion sample were immediately transferred into a 15 mL graduated test tube which was tightly sealed with a plastic cap and then stored at room temperature. After storage (1 h), a number of emulsions separated into an opaque (cream) layer at the top and a turbid or transparent (serum) layer at the bottom. Creaming stability was calculated using the following equation:

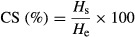
(4)where *H*_s_ is the height of the serum layer and *H*_e_ is the total height of the emulsion.

### Gelling ability

The least gelling concentration (LGC) of the plant flours was determined according to the method of Sathe and Salunkhe (1981). Different amounts of plant flours were weighed into test tubes containing 5 mL of 10 mmol/L Na_2_HPO_4_–NaH_2_HPO_4_ buffer (pH 4.0, 7.0, and 10.0) to make dispersions ranging in concentration from 20 to 200 g L^−1^. The samples were vortexed at room temperature for 30 min and the tubes were sealed and heated at 100°C in a water bath for 60 min. The tubes were cooled immediately under tap water and stored at 4°C overnight. To determine whether the suspensions had formed a gel the tubes were inverted. A firm gel was presumed to have been formed when on inverting the tube the dispersions did not flow, whereas the semi-solid structure that flowed somewhat on inversion was presumed to be a weak gel. The LGC was determined as the critical concentration below which no firm gel can be formed.

### Foaming capacity and stability

Foaming properties were determined according to the method of Shahidi et al. (1995) Sample (1.0 g) was added to 50 mL of 10 mmol/L Na_2_HPO_4_–NaH_2_HPO_4_ buffer (pH 4.0, 7.0, and 10.0) and the dispersions were allowed to hydrate for 15 min. The samples were whipped for 1 min at maximum speed using a Duronic DM300 mixer (Shinemart Ltd., Romford, UK) and the total volumes were recorded at 0 and 60 min. Foam ability was expressed as foam expansion at 0 min and foam stability was expressed as foam expansion after 60 min. Foam expansion was calculated from the following equation:

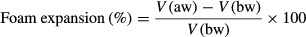
(5)where *V* (aw) is the volume (mL) after whipping and *V* (bw) is the volume (mL) before whipping.

### Water holding capacity

Water binding capacity of the plant flours was determined according to a slightly modified version of the method described by Beuchat (1977). Samples (1 g) were weighed and dispersed in 10 mL of 10 mmol/L Na_2_HPO_4_–NaH_2_HPO_4_ buffer (pH 4.0, 7.0, and 10.0) and placed in 50 mL centrifuge tubes. The dispersions were stirred on vortex at room temperature for 30 min and were then centrifuged at 3148*g* for 30 min. The supernatant was discarded and the tube was weighed. Water holding capacities were expressed as gram of water retained per gram of sample and was calculated using the following equation:

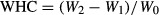
(6)where *W*_0_ is the weight of the dry sample, *W*_2_ is the weight of the tube plus sediment, and *W*_1_ is the weight of the tube plus dry sample.

### SDS-PAGE

Polyacrylamide gel electrophoresis (SDS-PAGE) was carried out on the plant flour dispersions according to the method described by Laemmli (1970) using a Mini-Protean 3 electrophoresis cell unit (Bio-Rad). Gel electrophoresis was run on a 4–20% Mini-Protean® TGX™ precast gel. The migration buffer contained 25 mmol/L Tris, 192 mmol/L glycine, and 0.1% SDS (pH 8.3). Flours were dispersed in dH_2_O (250 g L^−1^) for 30 min were centrifuged at 6708 *g* for 5 min. Supernatants were diluted 1× with dH_2_O and were then dispersed in an equal volume of sample buffer. 2-Mercaptoethanol (50 mL L^−1^) was added as reducing agent to the sample buffer (31.5 mmol/L Tris-HCl, pH 6.8, 10% glycerol, 1% SDS, 0.005% bromophenol blue). Samples were heat-denatured at 100°C for 2 min and 10 *μ*L of each sample were loaded on the gel. Electrophoretic migration was performed at 200 V (constant) for 40 min. The gel was stained with Coomassie Brilliant Blue R-250 staining solution for 1 h with gentle agitation and destained with Coomassie Brilliant Blue R-250 destaining solution for 2 h. The gel was scanned with a GS-800™ calibrated densitometer (Bio-Rad).

### Statistical analysis

All the data were averaged from at least three repeats coming from three different batches of samples and are reported as means and standard deviation. Statistical analyses were performed using one-way ANOVA to detect significant differences between samples (IBM SPSS statistics 22, Armonk, NY). *P* < 0.05 was considered significant.

## Results and Discussion

### Protein solubility

Protein solubility is a critical factor for the applicability of certain protein ingredients in functional food matrices. It is an important determinant of the sensory quality attributes of foods and impacts on application functionalities such as emulsifying ability and foam forming capacity (Kinsella 1976; Morr 1990).

**Figure 1:**
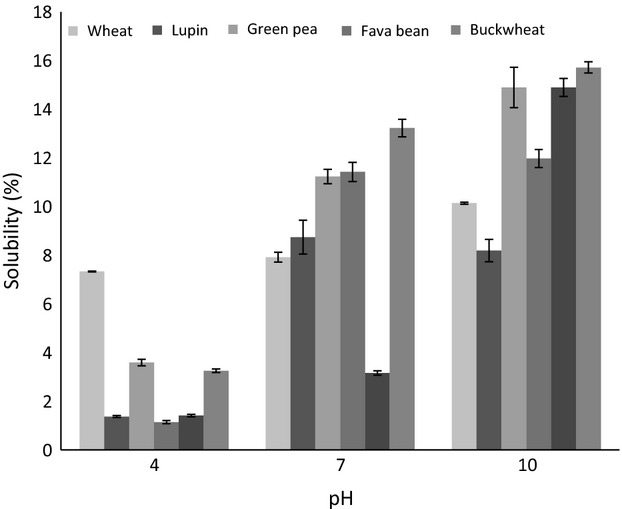
Solubility of flours at pH 4, 7, and 10. Results are presented as mean ± SE for triplicate analyses.

The protein solubility profiles of all the flours used in the present study were clearly pH dependent increasing over a pH range from 4 to 10 (Fig.1). Similar findings have been reported for other flours (Adebowale and Lawal 2004; Ma et al. 2011; Sreerama et al. 2012; Sridaran et al. 2012). At pH 4, which is near the isoelectric point of most proteins, protein–protein interactions are favored because of negligible molecular repulsion. Formation and subsequent precipitation of large molecular weight aggregates may arise, thus reducing protein solubility. Greater protein solubility above the isoelectric point at higher pH is likely associated with increased negative charge, ionic hydration, and electrostatic repulsion (Lawal 2004; Moure et al. 2006). In the present study, wheat flour proteins were the most soluble at pH 4. However, with the exception of hemp (pH 7) and lupin (pH 10) within the pH range 7–10 the protein content of wheat flour was less soluble than most other samples. Hemp, buckwheat, and green pea flours had the highest increase in solubility at pH 10. In contrast, there was a relatively small and steady increase in protein solubility of wheat flour in response to pH.

### Emulsifying properties

**Figure 2:**
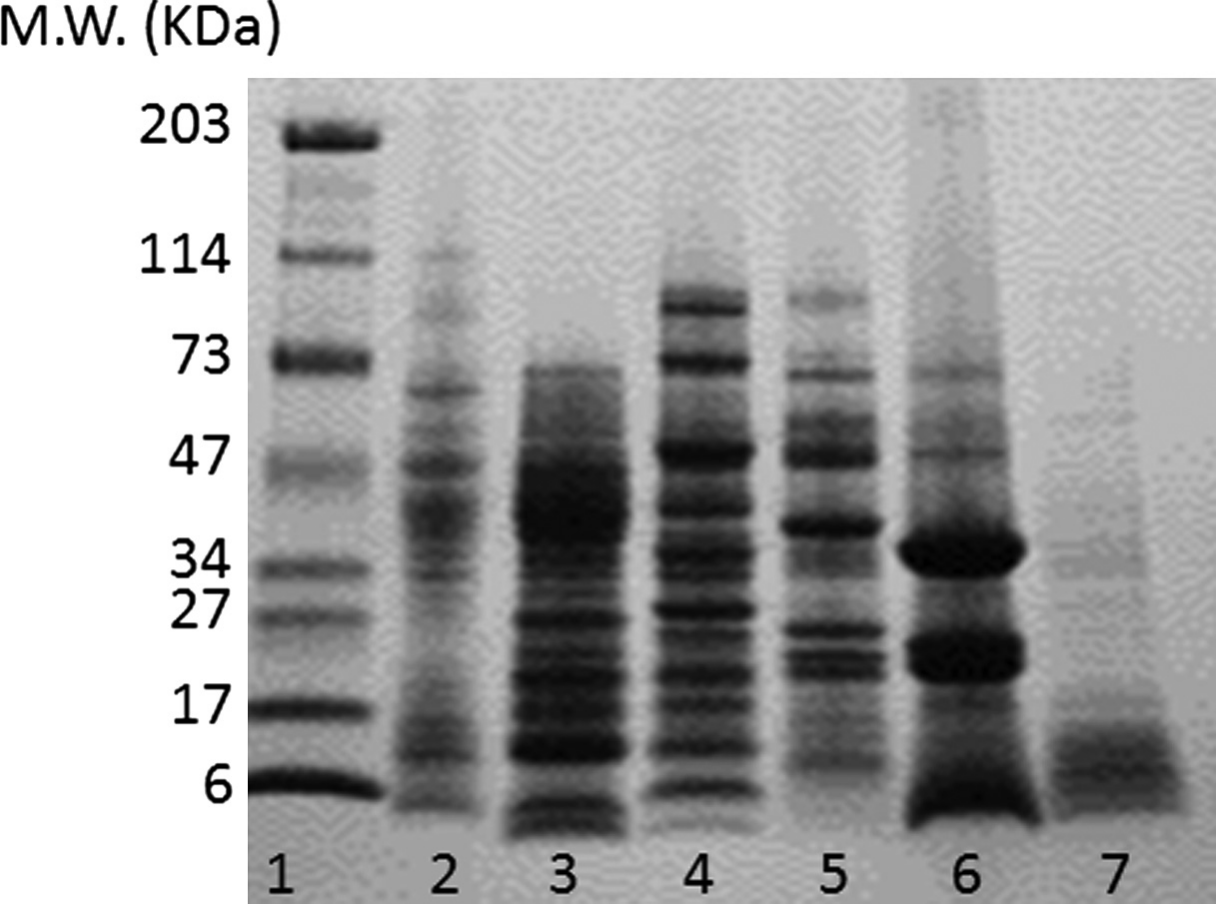
Electrophoretic migration patterns (SDS-PAGE) of protein dispersions prior to emulsion formation. Lane 1: molecular weight standards; lane 2: wheat; lane 3: lupin; lane 4: green pea; lane 5: fava bean; lane 6: hemp; lane 7: buckwheat. SDS-PAGE, sodium dodecyl sulfate polyacrylamide gel electrophoresis.

**Figure 3:**
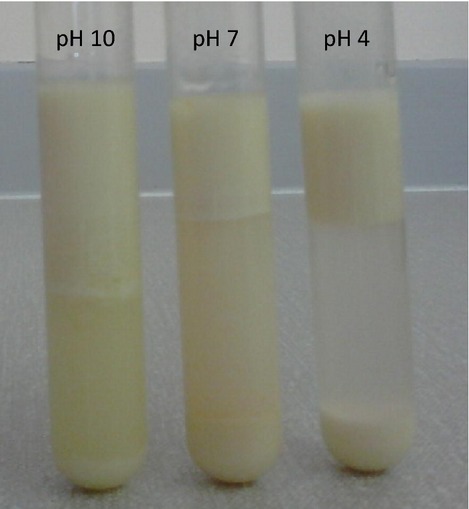
Effect of pH on creaming stability of oil-in-water emulsions (lupin) standing at room temperature for 24 h.

The emulsification process depends on the ability of proteins to adsorb rapidly at the oil–water interface where they form a strong, viscoelastic film around the oil droplets. This offers a degree of protection against any destabilizing mechanisms (Pearce and Kinsella 1978). Denaturation and partial unfolding of protein molecules upon adsorption at the interface with appropriate hydrophobic and hydrophilic orientation is critical for emulsion formation and stabilization (Carvalho et al. 2006). In the present study, the emulsifying ability and stability of all flour dispersions increased with increasing pH (Table2) despite marked differences in protein concentrations (Table1). Furthermore, as revealed by the migration pattern of SDS-PAGE, the protein composition of the different flours differs to a great extent (Fig.2). The lanes corresponding to lupin and green pea contain higher number of bands compared to the other samples. The intensity of the protein bands is also indicative of the protein concentration and results are in agreement with the macronutrient composition of the flours. Lanes 2 (wheat) and 7 (buckwheat) appear to have less protein compared to all other lanes. Thus, both qualitative and quantitative differences in protein content are expected to contribute to the emulsifying properties of each flour sample. In agreement with other studies, emulsifying ability and stability was highest at pH 10 and followed a similar pattern to the observed pH-dependent solubility (Adebowale and Lawal 2004; Sridaran et al. 2012). Solubility affects the ability of protein molecules to diffuse fast and adsorb at the interface. Such enhanced emulsifying properties at alkaline pH may also arise from the dissociation and partial unfolding of globular proteins. Resulting exposure of hydrophobic amino acid residues consequently increases the surface activity and adsorption at the oil and water interface (Nir et al. 1994). The pH-driven effect on emulsion stability had an impact on the creaming rate of the samples, which was noticeably lower at pH 10 (Fig.3). Wheat and lupin were the least promising emulsifying agents, whereas buckwheat and hemp had the highest emulsifying ability and stability indices at pH 10. Although adequate protein concentration is a prerequisite for emulsion formation and stabilization, the type of protein is also critical in terms of the reduction in the interfacial tension and the formation of a protective layer around the oil droplet (Prinyawiwatkul et al. 1993). This is reflected in lupin flour, which although has the highest protein content (400 g kg^−1^) shows poor emulsifying properties. Not all protein molecules are as effective as emulsifiers and this is deduced from the significant differences in the emulsifying activity and stability indices of the flours analyzed in this study.

**Table 2:** Effect of pH on the physicochemical properties of oil-in-water emulsions.

Flour type	EAI (m^2^/g) pH			ESI (min) pH			CS (%) pH		
	4	7	10	4	7	10	4	7	10
Wheat	23.4 ± 1.3b*	24.5 ± 3.4b*	30.1 ± 1.4b•	33.1 ± 20.6a*	45.3 ± 16.3a*,•	84.4 ± 7.3a•	84.6 ± 3.0d•	76.2 ± 4.2e•	58.9 ± 0.4c*
Lupin	10.5 ± 1.9a*	13.7 ± 0.5a*	18.1 ± 1.1a•	63.4 ± 23.5a*	109.8 ± 4.4b*,•	156.8 ± 21.9ab•	64.4 ± 1.7c°	55.5 ± 2.1c•	38.7 ± 1.5a*
Green pea	11.8 ± 2.2a*	37.8 ± 0.6c•	40.7 ± 1.9c•	25.5 ± 3.9a*	100.6 ± 13.4b•	131.0 ± 39.5ab•	61.0 ± 2.6b	–	–
Fava bean	12.5 ± 1.2a*	23.5 ± 2.7b•	38.2 ± 2.7c°	33.6 ± 4.9a*	80.0 ± 25.2ab*,•	135.4 ± 27.8ab•	70..0 ± 2.8c*	61.4 ± 0.5d*	–
Hemp	8.5 ± 1.1a*	17.2 ± 1.7ab•	44.2 ± 0.2c°	24.2 ± 0.8a*	63.8 ± 3.3ab*	1278.0 ± 162.9c•	58.9 ± 2.1b•	46.9 ± 1.4a*	–
Buckwheat	19.4 ± 2.4b*	40.0 ± 1.70c•	71.9 ± 1.2d°	29.0 ± 3.2a*	76.1 ± 7.8ab*	389.1 ± 37.7b•	50.7 ± 2.8a•	51.9 ± 2.7b•	44.3 ± 2.0b*

Results are presented as mean ± SD for triplicate analyses. EAI, emulsifying activity; ESI, emulsifying stability.

Different symbols (*•°) denote significant differences (*P* < 0.05) within rows. Different letters denote significant differences (*P* < 0.05) within columns.

### Foaming capacity and stability

**Figure 4:**
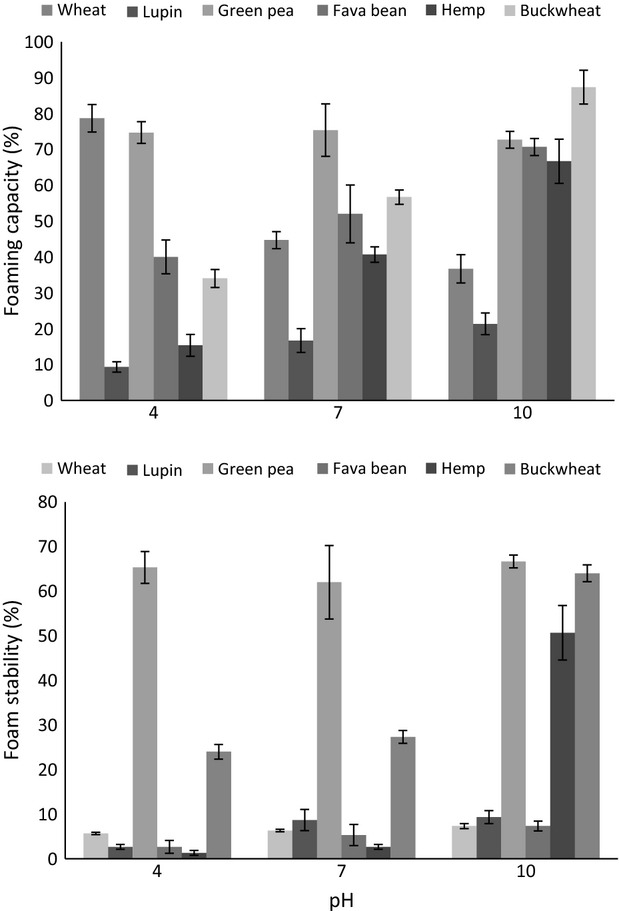
Effect of pH on whipping ability and foam stability of flours. Results are presented as mean ± SE for triplicate analyses.

The capacity of flours to form foams is widely exploited by the food industry for bakery and confectionery applications such as mousses, meringue cakes, and whipped toppings. Foaming capacity depends on the ability of proteins to adsorb quickly at the air–water interface during whipping, whereas foam stability is determined by the properties of the multilayer, cohesive film which surrounds the air bubbles and offers resistance against liquid drainage and droplet coalescence (Sreerama et al. 2012). The foaming properties of the flour samples differed significantly (Fig.4) possibly reflecting the influence of protein type and concentration on foaming capacity and foam stability (Kinsella 1979). Increasing pH in the range 4–10 enhanced the foaming properties of the flours, particularly for buckwheat and hemp. Olawuni et al. (2013) reported the improvement of foaming capacity for full fat and defatted Asparagus beans flours with increasing pH (4–12). This may be attributed to the increased solubility within the specified pH range, because foaming capacity requires rapid adsorption of protein at the air–water interface during whipping, penetration into the surface layer, and structural reorganization at the interface (Were et al. 1997). Furthermore, the improved ability to trap air particles at a pH far from the isoelectric point could be due to the increased flexibility and surface activity of the highly charged protein molecules (Aluko and Yada 1995). However, not all samples showed the same pH-dependent pattern of foaming properties. Green pea produced relatively thick, voluminous foams which were stable even at pH 4 (65%). In addition, the foaming capacity of wheat flour deviated from the theoretical expectation that solubility is positively correlated with whipping ability (Nakai 1983). Wheat proteins had maximum foaming ability at pH 4 (78.7%) followed by a decline in pH 7 (44.7%) and pH 10 (36.7%). Furthermore, despite high foaming abilities of wheat flour and fava bean flour at pH 4 and 10, respectively, stability was poor (5.7% and 2.7%, respectively). Foam stability is an important property because the usefulness of whipping agents depends on their ability to maintain the whip for as long as possible (Lin et al. 1974). In these cases, molecular flexibility appeared adequate to facilitate foam formation, but stability was compromised by intermolecular interactions at the interface.

### Gelling ability

A gel is a network between denatured molecules cross-linking to form aggregates containing large amounts of trapped water. The process of gel formation depends on several factors such as protein concentration, ionic strength, pH, and interaction with other components (Yasuda et al. 1986). Gelation is often a desired property in foods such as jellies, puddings, and in many meat and dessert applications. LGC is a measure of the ability of proteins to form a gel; a lower LGC suggests a better gelling capacity. The gelling behavior of the flours varied and was also dependent on pH (Table3). The majority gelled within a concentration range of 100–140 g L^−1^. Other studies indicate that 120 g L^−1^ is the minimum concentration for dehulled cowpea seed and bitter lupin seed flours to form a gel (Khalid and Elharadallou 2013). Wheat, green pea, buckwheat, and fava bean were the most capable of forming firm gels compared with lupin and hemp. This indicates that protein content is not the only determinant of LGC. Hemp flour, even at 200 g L^−1^ concentration, produced a paste rather than a cohesive gel, suggesting that the intensity of intermolecular interactions was too weak to overcome repulsive forces. Flours with lowest LGC (wheat and buckwheat) also had the highest carbohydrate content (Table1) supporting the view that gelation may be also affected by the relative ratios and interactions of nonprotein components such as polysaccharides and lipids (Sathe et al. 1982). Carbohydrates are reported to decrease the thermodynamic affinity of proteins for water molecules and magnify the magnitude of interaction between the protein molecules, thus improving the gelling capacity (Adebowale and Adebowale 2008). The process of gelation is also affected by pH, which can alter the charge distribution among the amino acid side chains and can either decrease or increase the protein–protein interactions (Raikos et al. 2007). At pH values far from the isoelectric points, the protein surface charge is increased and thus significant repulsive forces inhibit protein–protein interactions resulting in decreased gelling capacity (Elofsson et al. 1997). This effect could account for the inability of lupin flour to form a gel at pH 10 and the higher LGCs of buckwheat flour at pH 7 and 10.

**Table 3:** Effect of pH on the gelling behavior of flours at different concentrations.

Flour concentration (% w/v)	Wheat pH			Lupin pH			Green pea pH			Fava bean pH			Hemp pH			Buckwheat pH		
	4	7	10	4	7	10	4	7	10	4	7	10	4	7	10	4	7	10
2	××	××	××	××	××	××	××	××	××	××	××	××	××	××	××	××	××	××
4	××	××	××	××	××	××	××	××	××	××	××	××	××	××	××	××	××	××
6	××	××	××	××	××	××	××	××	××	××	××	××	××	××	××	××	××	××
8	××	××	√±	××	××	××	××	××	×±	××	×±	××	××	××	××	√±	××	××
10	××	××	√√	××	××	××	×±	×±	±±	×±	±±	×±	××	××	××	√√	××	×±
12	×±	√√	√√	××	××	××	√√	√√	√√	±±	√√	±±	××	××	××	√√	±±	±±
14	√√	√√	√√	××	√√	××	√√	√√	√√	√√	√√	√√	××	××	××	√√	√±	√√
16	√√	√√	√√	××	√√	××	√√	√√	√√	√√	√√	√√	××	××	××	√√	√√	√√
18	√√	√√	√√	×±	√√	××	√√	√√	√√	√√	√√	√√	××	××	××	√√	√√	√√
20	√√	√√	√√	√√	√√	×±	√√	√√	√√	√√	√√	√√	××	××	××	√√	√√	√√

×, no gel; ±, weak gel; √, firm gel; √√, least gelling concentration.

### Water holding capacity

**Figure 5:**
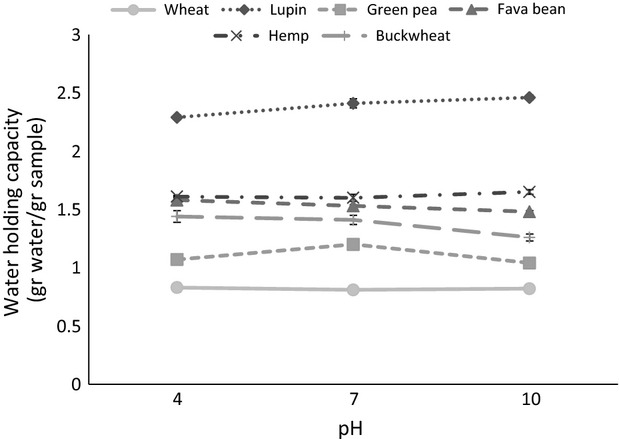
Effect of pH on water binding capacity of flours. Water binding capacity is expressed as gram of water retained per gram of sample. Results are presented as mean ± SE for triplicate analyses.

WHC is the ability of a food product to physically hold water against gravity (Kinsella 1979). It is an important property of flours which to a large extent determines their applicability as food ingredients. Hence flours with high WHC are widely used in meat products, custards and soups to enhance body thickening and viscosity, and in baked products to improve freshness and handling characteristics (Wolf 1970). The WHC of the flours in the present study was not affected by pH (Fig.5). However, significant differences were observed between the water binding properties of the different types of flours and followed the order lupin>hemp>fava bean>buckwheat>green pea>wheat. As protein content appears to be a critical determinant of the ability of the flours to imbibe water, the WHC of lupin and hemp may be a reflection of their high protein content (Table1). Similar WHC (1.34 mL/g) for lupin flour has been previously documented by other researchers (Khalid and Elharadallou 2013). Accordingly, the decreased ability of wheat flour to bind water may be attributed to the low protein content (129 g kg^−1^) of this sample. Furthermore, other factors such as the polar to nonpolar amino acid ratios may influence WHC as polar amino acid residues have an affinity for water molecules (Zayas 1997). However, effects of hydrophilic carbohydrates such as polysaccharides on WHC are unlikely (Kaur et al. 2007).

## Conclusions

The functional properties of hemp, buckwheat, fava bean, green pea, and lupin flours indicate that they could contribute desirable attributes to a wide range of food products. The functional properties of the flours are dependent on pH and can be suitably modified to achieve desired qualities in new food products. Variations in functional properties between the flours under investigation are attributed to differences in protein type and content in addition to the carbohydrate concentration of the flours. Buckwheat and hemp exhibited promising emulsifying and foaming properties at alkaline pH. Green pea and fava bean showed good gelling abilities over a wide pH range and lupin showed good water binding capacity. Furthermore, the high protein content of these underutilized flours suggests that they could serve as cheap and alternate source of proteins. These favorable nutritional and functional properties of flours could be exploited in the preparation and development of food products, such as bakery and confectionery products, sauces and dressings, soups, meat products, and others. The flours from these plant sources may also be suitable for producing composite flours as partial substitutes of wheat flour in diverse food products. Further studies are required to investigate protein functionality of these flours in composite flours and in food systems.
